# The impact of CyberKnife's prescription isodose percentage on intracranial target planning

**DOI:** 10.1120/jacmp.v15i5.5081

**Published:** 2014-09-08

**Authors:** Sung‐Woo Lee, Sunyoung Jang, Anil P. Pyakuryal, Kenneth Chang, Terence T. Sio

**Affiliations:** ^1^ Alpert Medical School of Brown University Providence RI; ^2^ Department of Radiation Oncology Rhode Island Hospital Providence RI; ^3^ Princeton Radiation Oncology Jamesburg NJ; ^4^ Radiation Epidemiology Branch Division of Cancer Epidemiology and Genetics, National Cancer Institute Bethesda MD; ^5^ Department of Radiation Oncology Mayo Clinic Rochester MN USA

To the Editor:

Recently, a detailed comparative study regarding intracranial Gamma Knife (GK) vs. CyberKnife (CK) intracranial dosimetry has been published by your Journal.^(1)^ In a group of 15 patients with 26 brain metastases, we showed that CK produced more homogeneous and conformal plans, while the GK plans had sharper peripheral dose falloff in most cases. In the CK plans, by convention, the applied range of prescription isodose percentage (PIP) was 77%–92%, with a median value of 86%. Intrigued by the results, we hypothesized that lowering the PIP in CK planning would improve peripheral dose falloff, without compromising the excellent dosimetric conformality which had previously been achieved. Secondarily, it was expected that, as PIP decreased, the stereotactic radiosurgical (SRS) plans would become less homogeneous as maximum dose within the target increased.

We thank you for the opportunity to share with you the additional results that were generated from this investigation. Parts of the methods and materials have been previously described.^(1)^ We compared the relative dosimetric merit of various prescription isodose levels in CK's MultiPlan (Accuray Inc, Sunnyvale, CA). The same 15‐patient series was used for dosimetric planning. For each tumor, the PIP was varied at three levels averaging approximately 50, 65, and 85% (CK50, CK65 and CK85; Table 1). The homogeneity (HI) and gradient (GI) indices, modified conformity index (mCI, the ratio of the prescription isodose volume to the tumor volume receiving at least the prescription dose), and an MPS‐defined quantity called “new CI” (nCI, the ratio of mCI to target coverage, also the inverse of van't Riet's Conformation Number) were computed. For peripheral dose falloff, GI50 was calculated as the ratio of the volume enclosed by the isodose at 50% of the prescription dose level to the volume enclosed by the original prescription isodose. GI25, GI40, GI60, and GI80 were calculated in a similar manner. Statistical analyses were performed using analysis of variance (ANOVA) and nonparametric Kruskal‐Wallis tests.

We found that the mean tumor volume was 4.4 cm^3^; a median dose of 18 Gy was prescribed. For CK50, CK65, and CK85 series, the coverage was maintained at 96%–100% in all cases. Optimized plans in each scenario across various PIPs were computed, and dosimetric constraints of critical organ structures were all met. Minimum, average, maximum doses, HI, nCI, GI25, GI50, and MU were reported (Table 2). Comparing across the CK50, CK65, and CK85 series, the median mCIs were: 1.48, 1.36, and 1.52, p=.086, respectively. The remaining gradient indices were: GI40 (5.8, 6.9, and 7.6, p=0.0008); for GI60 (2.8, 3.4, and 3.8), GI80 (1.6, 1.9, and 2.2), and GI90 (1.3, 1.4, and 1.6), p<0.00001 in all cases. In our study, as expected, the selection of a PIP had a statistically significant impact on HI, mean, and maximum doses. By both mCI and nCI, CK65 produced the most conformal plans which nearly reached statistical significance. Importantly, CK50 had significantly sharpest dose falloff at all gradient index levels, with the exception of GI25. However, dosimetric plans prescribing to CK50 required significantly longer treatment times by MU estimation.

In current clinical practices, deciding a prescription isodose level in CK varies by individual plan and preference, and clearly no consensus exists. The CK is a relatively new modality for SRS, which is stereotactically capable for extracranial indications, as well. For GK and linac‐based intracranial SRS, it is common to prescribe to 40%–60% and 80%–95% of PIP, respectively. It is then customarily believed that the PIPs of CK should fall between those of the GK and linac‐based SRS plans, as CK shares features of both. For CK dosimetric planning, some authors used median PIPs of about 79%,^(2)^ while others believed values near 73% may be more appropriate.^(3)^ Inoue et al.^(4)^ used 60% PIP for a three‐fractioned course of 27 Gy. Our current data evaluated a wider range of PIPs compared to these published studies. Additionally, some researchers^(5)^ tried to compare different radiosurgery modalities by interpreting the equivalent uniform biologic effective dose of normal brain tissue for GK, CK, and linac‐based systems, with largely equivalent results. Petti et al.^(6)^ suggested dosimetric guidance for GK users to optimize CI to reduce normal brain toxicity derived by empirical formula. According to their study, CI was an important dosimetric parameter to consider when tumor size exceeded 1 cm^3^, which is equally important in CK planning.

**Table 1:** Tested patient's tumor location, volume, and PIP

			*Final PIP Chosen (%) CK65*		
*Patient No*.	*Tumor Location*	*Volume of Tumors (cm* ^*3*^ *)*	*CK50*	*CK65*	*CK85*
1	Left Frontal	1.2	50	64	88
	Right Cerebellar	1.4	50	68	78
	Vermis	1.7	50	66	79
	Left Occipital	2.1	50	68	81
2	Right Frontal	1.2	50	69	87
3	Right Parietal	0.3	51	68	86
4	Right Cerebellar	7.6	50	65	90
5	Right Frontal	5.9	52	65	86
6	Left Temporal	0.1	50	69	88
7	Left Parietal	2.9	53	68	89
	Parietal Fossa	4.8	48	65	88
8	Left Anterior Frontal	19.9	50	66	91
	Left Post Frontal	5.1	52	70	92
9	Left Cerebellar	7.0	50	65	83
	Left Frontal	1.2	50	65	77
10	Vermis	5.8	50	66	85
	Right Cerebellar	11.5	49	65	84
11	Right IAC	0.5	52	68	91
	Middle Cerebellar	0.7	50	69	88
12	Right Frontal	2.1	50	69	81
	Left Temporal	0.4	52	68	83
	Right Frontal	4.6	54	65	82
13	Right Post Frontal	3.6	50	65	83
14	Vermis	14.5	49	66	82
	Brainstem	1.5	50	65	89
15	Right Frontal	8.9	48	65	79
Median (Range)		2.5 (0.1–19.9)	50 (48–54)	66 (64–70)	86 (77–92)

PIP=prescription isodose percentage; CK=CyberKnife; IAC=internal auditory canal.

**Table 2:** Summary of CyberKnife's dosimetric parameters (mean values) as varied by prescription isodose levels

	*CK50*	*CK65*	*CK85*	*F(H)‐ratio*	*p‐value*
Minimum Dose (Gy)	17.6	17.3	17.6	0.1	0.92
Mean Dose (Gy)	27.1	22.9	20.5	30.8	<0.00001 ^a^
Maximum Dose (Gy)	36.9	27.9	22.1	102.5	<0.00001 ^a^
Homogeneity Index (HI)	1.99	1.50	1.18	1568.1	<0.00001 ^a^
New Conformity Index (nCI)	1.50	1.38	1.55	2.6	0.09
Gradient Index 25 (GI25)	15.6	16.7	16.3	0.5	0.80
Gradient Index 50 (GI50)	3.8	4.7	5.2	22.3	<0.0001 ^a^
Monitor Unit (MU)	20084	13952	12025	12.4	0.0021^a^

a Statistically significant (two‐sided alpha=0.05).

CK=CyberKnife.

In terms of dose falloff, as Sahgal et al.^(7)^ and Ma^(8)^ reported, the choice of PIP was an important factor to consider in plan quality optimization. Particularly, the authors noted that, in patients with multiple metastases, the choice of PIP became critical as SRS treatment extended to increased number of target sites. In our study, we also observed similar peripheral dose falloff variations as a result of different PIPs. Specific to CK, the multiplan system (MPS) has three optimization functions to emulate GK‐like “isocentric” forward planning, in additional to IMRT‐like multi‐isocentric “conformal” and “sequential” inverse planning algorithms; the optional selection of such a dosimetric function, especially in relation to varying PIP, should be the topic of a future study.

We conclude that, for CK dosimetric planning in focused irradiation of intracranial targets, the choosing of a PIP had a statistically significant impact on HI, minimum, mean, and maximum doses. By both mCI and nCI criteria, CK65 produced the most conformal plans which nearly reached statistical significance. Although CK50 plans required longer treatment times as estimated by MU, their peripheral dose falloff was sharpest at all gradient index levels, except for GI25. PIP is an important variable to consider in CK planning; tradeoff among homogeneity, treatment time, and dose gradient falloff must be carefully balanced in individualized stereotactic radiosurgical plans.
